# Uncertainty about amplitude eliminates negative masking in a pure-tone amplitude discrimination experimenta)

**DOI:** 10.1121/10.0003042

**Published:** 2021-01

**Authors:** Christopher Conroy, Gerald Kidd

**Affiliations:** Department of Speech, Language and Hearing Sciences and Hearing Research Center, Boston University, 635 Commonwealth Avenue, Boston, Massachusetts 02215, USA cwconroy@bu.edu, gkidd@bu.edu

## Abstract

The role of uncertainty and its reduction in producing the “negative masking” of amplitude increments that is often observed in pure-tone amplitude discrimination experiments using circathreshold pedestals was investigated. It was found that negative masking is eliminated by uncertainty induced by roving the pedestal level across trials. On the basis of this finding, as well as those from a previous study, it is argued that, consistent with a longstanding theory of negative masking based on the notion of “intrinsic uncertainty,” negative masking requires near-optimal stimulus conditions, under which uncertainty about increment parameters is more or less absent.

## 1. Introduction

On each trial of a typical, two-interval, two-alternative forced-choice (2I-2AFC) pure-tone amplitude discrimination experiment, the observer is presented with two brief tones differing only in amplitude: a “pedestal” of amplitude *A* and that pedestal plus an “increment” of amplitude Δ*A*. The observer is then asked to make a judgement regarding which tone had the greater overall amplitude. Typically, the experimenter is concerned with finding the smallest value of Δ*A* for which the observer can discriminate a difference between the two tones on some specified proportion of trials. For a given *A*, this value is denoted the “just noticeable difference” (JND) in amplitude, Δ*A_JND_*. Weber's law states that Δ*A_JND_* = *kA*, where *k* is a constant and is commonly referred to as the “Weber fraction” (Fechner, 1860). When the pedestal and the increment are pure-tones of sufficient duration and *A* exceeds the observer's “absolute detection threshold,” *A_0_*, Weber's law more or less holds true, save for a slight decrease in *k* with *A*. This decrease in *k* with *A* is the well-known “near-miss to Weber's law” (McGill and Goldberg, 1968).

Inasmuch as the increment can be considered “the signal” and the pedestal “the masker,” pure-tone amplitude discrimination essentially is equivalent, both practically and conceptually, to masked detection. By this view, the plot of log Δ*A_JND_* vs log *A* is a “masking function” (Miller, 1947). Weber's law predicts a masking function that is a straight line with a slope of one. The near-miss to Weber's law, then, which yields a slope of slightly less than one (e.g., McGill and Goldberg, 1968; Green *et al.*, 1979; Hanna *et al.*, 1986; Viemeister and Bacon, 1988; Shepherd and Hautus, 2009), is broadly consistent with this prediction. Nevertheless, explaining why the slight deviation occurs has been a core topic in auditory research for nearly a century (cf. Reisz, 1928). Curiously, however, a phenomenon that runs directly counter to the predictions of Weber's law, “negative masking” (Raab *et al.*, 1963), has received comparatively little attention.

Whereas the near-miss shows that, for *A* > *A_0_*, Δ*A_JND_* monotonically increases with *A*, for *A* in the region near *A_0_*, a distinct, nonmonotonic “dip” in the masking function is apparent: as *A* increases from some very low level below *A_0_*, Δ*A_JND_* first *decreases* to a minimum value, where Δ*A_JND_* < *A_0_*, before increasing in turn (e.g., Hanna *et al.*, 1986; Viemeister and Bacon, 1988; Shepherd and Hautus, 2009; Shepherd *et al.*, 2013; Conroy *et al.*, 2020). This improvement in detection performance under “masked” *re.* unmasked conditions is negative masking. In the auditory literature,^1^ various theories of negative masking have been proposed (see Shepherd and Hautus, 2009, for a review). Currently, however, there is no consensus regarding which best accounts for the available data. In a recent study (Conroy *et al.*, 2020; hereafter, “the previous study”), we tested the predictions of two competing theories of negative masking, one based on nonlinear transduction in the auditory system (e.g., Hanna *et al.*, 1986), the other on “intrinsic uncertainty” (e.g., Tanner, 1961), and found evidence in support of the latter. We extend that work here, focusing in particular on whether negative masking is obtained under conditions of high observer uncertainty induced by random variations in the amplitude of the pedestal.

The nonlinear transducer hypothesis posits that a “dipper”-shaped masking function exhibiting negative masking, i.e., a masking function with a circathreshold dip and suprathreshold “handle” governed by Weber's law, or something near to it (Solomon, 2009), simply maps the underlying transducer function. The transducer is accelerating at low pedestal levels and compressive at higher pedestal levels. The accelerating portion yields the dip and negative masking (e.g., Hanna *et al.*, 1986), whereas the compressive portion yields the handle and the near-miss (e.g., Fechner, 1860; Durlach and Braida, 1969; Hanna *et al.*, 1986). The intrinsic uncertainty hypothesis, on the other hand, posits that, when *A *=* *0, the observer possesses a large “intrinsic uncertainty” (e.g., Green, 1961) about which channel is most sensitive to the increment and therefore monitors many irrelevant channels when forming a detection decision (e.g., Tanner, 1961). Each channel is contaminated by internal noise, and when Δ*A* is small, this noise impedes detection. That is, on a significant proportion of trials, the noise in at least one of the many irrelevant channels will produce a sensation that exceeds the sensation produced by the increment. This yields a “false alarm,” and inflates *A_0_* in turn (Pelli, 1985). When *A* is small but nonzero, however, and is small enough so as to preclude masking, the pedestal serves to focus the observer's attention on the single relevant channel, rendering such noise sensations moot. With the aid of the pedestal “cue,” detection improves, and negative masking is obtained. Note that, for *A* ≫ *A_0_*, the noise in the irrelevant channels will only rarely exceed the sensation produced by the stimulus, and thus uncertainty is irrelevant in the suprathreshold region. Note also that the relevant/irrelevant channels are conceptualized as being tuned along the dimension of the uncertainty, whatever that dimension may be.

In formulating the intrinsic uncertainty hypothesis, Tanner (1961) speculated that, in the absence of the pedestal cue, the observer may be uncertain about a multitude of stimulus parameters, including the frequency, phase, starting time, duration, and/or amplitude of the increment. By this view, only when all sources of uncertainty are minimized, does negative masking obtain. In the previous study, we focused on the effects of uncertainty about frequency. Uncertainty about frequency was induced by randomly varying the frequency content of a (nonpedestal) masker from interval to interval on each trial. We found that, under such conditions, the hypothesized attentional benefit of the pedestal cue was thwarted, and negative masking eliminated. In other words, we “added frequency uncertainty back in” to a pure-tone amplitude discrimination experiment, presumably “forcing” the observer to monitor some number of irrelevant frequency channels on each trial and eliminated negative masking in the process. We took this finding as evidence in support of the intrinsic uncertainty hypothesis, as opposed to a nonlinear transducer, given that the nonlinear transducer hypothesis does not admit a role for such attentional effects. In this study, we took a conceptually similar approach, but focused instead on the effects of uncertainty about amplitude. Uncertainty about amplitude was induced by “roving” the pedestal level across trials. (Note, however, that the pedestal level was the same across intervals of each trial.) In some ways, this may be considered a more direct test (than adding masker frequency uncertainty) of the intrinsic uncertainty hypothesis because the stimulus variability is imposed only along the acoustic dimension that forms the dependent variable in the task. It was hypothesized that, as in the case of uncertainty about frequency, uncertainty about amplitude would eliminate negative masking—consistent with the intrinsic uncertainty hypothesis, but not with a nonlinear transducer.

Implicit in this hypothesis is the notion that uncertainty about amplitude manifests as a failure of selective attention applied over an underlying array of “amplitude channels,” and that each of these channels is corrupted by an independent source of internal noise. Also implicit is the notion that all forms of uncertainty (or at least amplitude and frequency uncertainty) are “the same” in the sense that they are all equally disruptive to detection and/or discrimination performance, and therefore will have comparable effects on negative masking. While it is easy to conceive of such a failure of attention across frequency channels, and the effects on negative masking of added noise therein, it is perhaps not as easy to conceive of such a failure across amplitude channels, or even the existence of such channels at all. Indeed, alternative conceptualizations of the effects of the pedestal rove in terms of a more general increase in internal noise are possible, which do not require recourse to such notions (e.g., Berliner and Durlach, 1973; Berliner *et al.*, 1977). Thus, in addition to examining the predictions of the nonlinear transducer and intrinsic uncertainty hypotheses in the context of negative masking, another motivation for the current study simply was to investigate the roles of uncertainty and selective attention in pure-tone amplitude discrimination experiments more broadly.

## 2. Methods

### 2.1 Observers

Three observers with normal hearing (including the first author, Obs 2) participated (three males, 20–30 yr). The same three observers participated in the previous study (Experiment 2), and some data obtained in that study were reanalyzed and/or reproduced here as reference values. Note that, to facilitate comparisons with the previous study, observers are numbered here starting with “2,” despite the fact that only three observers were tested.

### 2.2 Stimuli

Stimuli were generated digitally using matlab (MathWorks, Inc., Natick, MA) software at a sampling rate of 44100-Hz and were presented, via a 24-bit sound card (RME HDSP 9632, Haimhausen, Germany), to the observer's left ear using a pair of Sennheiser (Sennheiser Electronic GmbH and Co. KG, Wedemark, Germany) HD280 pro earphones. All stimuli (pedestals and increments) were pulsed, 100-ms, 1000-Hz pure-tones, including 5-ms cosine-squared onset-offset ramps.

### 2.3 Procedures

Observers completed the experiment individually while seated in a double-walled, sound-treated Industrial Acoustics Company (IAC, North Aurora, IL) booth. The task was 2I-2AFC pure-tone amplitude discrimination, as described in Sec. 1. Estimates of Δ*A_JND_*, i.e., amplitude discrimination thresholds, were obtained for each of six values of *A*. They were –9, 0, 9, 27, 45, and 81 dB *re. A_0_* [i.e., dB sensation level (SL)], where *A_0_* was unique to each observer and was as measured in the previous study. *A_0_* was 1.3, 11.3, and −1.8 dB sound pressure level (SPL) for Obs 2, 3, and 4, respectively.

A two-down, one-up adaptive tracking algorithm was used for stimulus placement. At the beginning of the experiment, four adaptive tracks consisting of 50 total trials were initiated for each *A*. These 24 tracks (four tracks × six *A*) then were interleaved across all subsequent trials of the experiment. This had the effect of introducing a random rove in pedestal level across trials. Trials were completed in blocks of 50 and consisted of the following sequence of events: a 545-ms warning light, a 545-ms pause, and two 100-ms observation intervals separated by a 545-ms interstimulus interval. Both observation intervals contained a pedestal of equal amplitude; one contained an increment; the interval that contained the increment was chosen at random on each trial. Feedback was provided on all trials.

Δ*A* was adjusted by adapting on 20log(Δ*A*/*A*). The initial step size was 4 dB; it was reduced to 2 dB following the first four reversals. Following data collection, thresholds were obtained for each *A* by pooling the results of each of the four adaptive tracks and fitting a psychometric function (cumulative Gaussian) to the data on coordinates of proportion correct vs 20log(Δ*A*/*A*) using the psignifit 4 toolbox for matlab (Schütt *et al.*, 2016). In fitting the psychometric functions, only data in the range 0.25 ≤ *d'* ≤ 3 were included. For each *A*, thresholds were obtained by extracting the point at which the fitted psychometric function crossed 0.71.

The net of this procedure was a “roving-level” masking function for each observer, i.e., a masking function obtained under “roving-level” conditions in which *A* varied randomly from trial to trial. As discussed in Sec. 1, this trial-by-trial variation in *A* was hypothesized to introduce (relative to “fixed-level” conditions such as those tested in the previous study) uncertainty about amplitude into the experimental situation, thereby counteracting the hypothesized attentional benefit of the pedestal cue and precluding negative masking. As such, it was of interest to make a comparison between these roving-level masking functions and the “fixed-level” masking functions obtained under fixed-level conditions in the previous study. In the previous study, however, the fixed-level masking functions were obtained by averaging the adaptive tracking data in the typical manner. To maintain consistency with the methods used here (thresholds based on psychometric function fits), the fixed-level data from the previous study were reanalyzed by fitting a psychometric function to the adaptive tracking data for each *A* in the manner just described, and “reanalyzed” thresholds were obtained on the basis of these fitted functions. Reanalyzed thresholds and those previously reported were quite similar (r = 0.95, p < 0.001). In what follows, reanalyzed values are reported in the text and included in the statistical analyses, but previously reported values also are shown in the figures for the sake of visual comparison.

## 3. Results

**Fig. 1.**
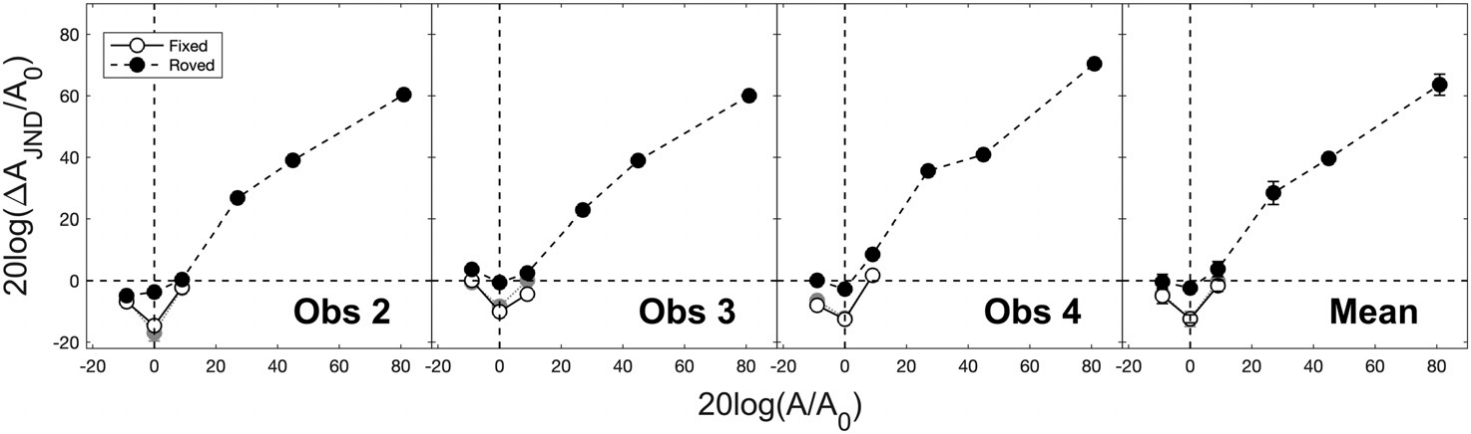
Roving-level masking functions (filled black symbols, dashed lines) for the three observers and the mean across observers. Also shown for comparison are the fixed-level masking functions obtained in the previous study for the same three observers and the mean across observers. Note that two different fixed-level masking functions are shown in each panel, representing two different methods for calculating thresholds: the open symbols (solid lines) give reanalyzed thresholds, whereas the filled grey symbols (dotted lines) give those previously reported (see text for details). Error bars give ±1 standard error of the mean.

The roving-level masking functions (filled black symbols, dashed lines) for the three observers and the mean across observers are shown in Fig. 1. Also shown for comparison are the fixed-level masking functions obtained in the previous study for the same three observers and the mean across observers. Two different fixed-level masking functions are shown in each panel: the open symbols (solid lines) give reanalyzed thresholds, whereas the filled grey symbols (dotted lines) give those previously reported. Note that both the ordinate and abscissa of each panel of Fig. 1 are normalized by *A_0_* and, as such, are in units of dB SL. Points that fall below the horizontal dashed line, then, indicate negative masking (Δ*A_JND_* < *A_0_*). Also note the wide range of amplitudes plotted along both axes, which, in order to capture the full roving-level masking function for each observer, spans a more than 100-dB range.

Perhaps the most obvious feature of the roving-level masking functions shown in Fig. 1 is the masking, i.e., the suprathreshold handle: for *A* > *A_0_*, Δ*A_JND_* increased with *A*, broadly in accordance with Weber's law. A straight line fitted to the thresholds (pooled across observers) for all *A* ≥ 9 dB *re. A_0_* (i.e., pedestals that, being ≫ *A_0_*, presumably were uncontaminated by uncertainty effects) had a slope of 0.79. This is the near miss to Weber's law. Neither nonlinear transduction nor intrinsic uncertainty predict an effect of the pedestal rove in the suprathreshold region and, as such, the handle *per se* is relatively uninformative with respect to evaluating these two hypotheses. One interesting aspect of the handle reported here, however, is the slope of 0.79, which is somewhat shallower then what has been reported previously for suprathreshold fixed-level masking functions obtained using similar stimuli, where values in the range 0.85 to 0.95 are more common (e.g., Green *et al.*, 1979; Hanna *et al.*, 1986; Viemeister and Bacon, 1988; Shepherd and Hautus, 2009). One possible reason for the difference is that the pedestal rove may have inflated thresholds for the mid-level pedestals relative to what “would have been obtained” under fixed-level conditions. For example, a slight bowing in the masking functions, driven largely by the mid-level pedestals, is apparent (e.g., Obs 4), reminiscent of the “mid-level hump” that often is observed in masking functions obtained using short-duration, high-frequency tones (e.g., Carlyon and Moore, 1984). Indeed, Pienkowski and Hagerman (2009) have shown that a pedestal rove can introduce a mid-level hump into the masking function where one typically is not apparent and, thus, this may have been a factor here.

**Fig. 2.**
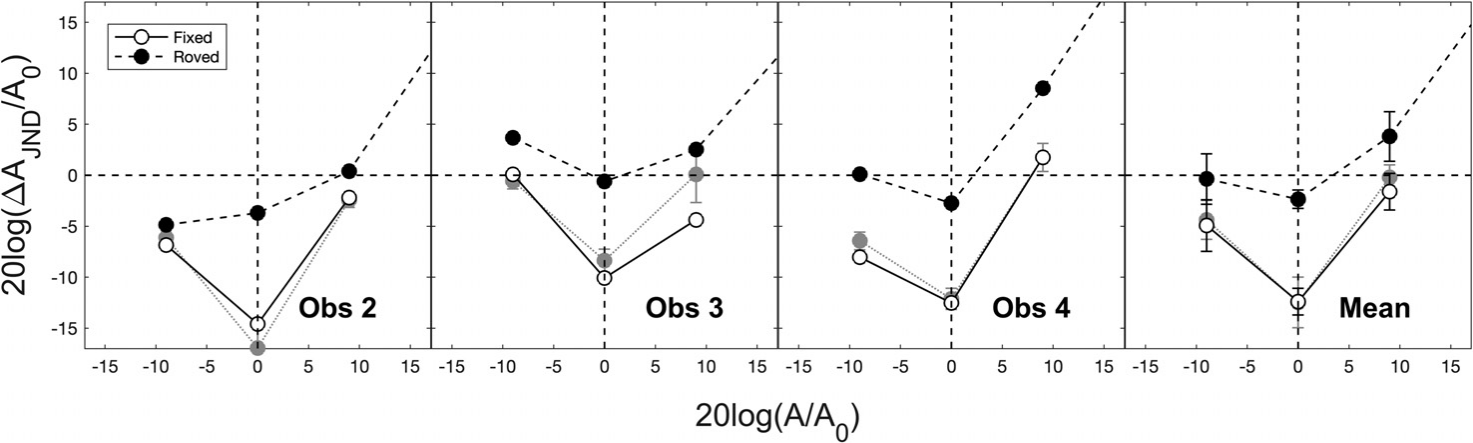
Close-up of the circathreshold region of the masking functions depicted in Fig. 1.

Of greater interest for the purposes of this study, however, was how uncertainty about amplitude affected the shape of the roving-level masking functions in the circathreshold region and the extent to which these functions exhibited the characteristic dipper shape and negative masking often observed in circathreshold masking functions obtained under fixed-level conditions (e.g., Hanna *et al.*, 1986; Viemeister and Bacon, 1988; Shepherd and Hautus, 2009; Shepherd *et al.*, 2013; Conroy *et al.*, 2020). The findings were clear: no dipper shape/negative masking was found for the roving-level condition. Whereas the fixed-level masking functions obtained in the previous study showed a dramatic dipper shape and large amounts of negative masking, uncertainty about amplitude all but eliminated the dip and eradicated negative masking. Under fixed-level conditions, negative masking (absolute detection minus discrimination threshold in dB SL) was at a maximum for *A* = *A_0_* and ranged from 8 to 17 dB across observers (mean = 12 dB, standard error = 2 dB). Under roving-level conditions, by contrast, negative masking was reduced dramatically, and ranged from 0 to 4 dB (mean = 3 dB, standard error = 1 dB). A two-way repeated-measures analysis of variance conducted on the thresholds for the three circathreshold pedestals revealed a significant main effect of uncertainty [fixed vs roved, F(1,2) = 177.29, p < 0.01] and pedestal level [–9, 0, or 9 dB *re. A_0_*, F(2,4) = 17.39, p = 0.01], and a significant two-way interaction [F(2,4) = 7.62, p < 0.05]. Post hoc t-tests (paired, p < 0.05) indicated that, of the three circathreshold pedestals, thresholds increased significantly under roved- *re.* fixed-level conditions for *A = A_0_* only and that, under roving-level conditions, negative masking was never significantly greater than zero. In other words, uncertainty about amplitude essentially smoothed out the dip in the dipper function for amplitude discrimination and eliminated negative masking. Figure 2 shows a close-up (i.e., a range along each axis of 30 dB rather than the 100 dB of Fig. 1) of the dip/no dip region of the masking functions shown in Fig. 1. Clearly, uncertainty had an effect.

## 4. Discussion

The primary purpose of this study was to examine the predictions of two competing theories of negative masking, one based on a nonlinear transducer (e.g., Hanna *et al.*, 1986), the other on intrinsic uncertainty (e.g., Tanner, 1961). The results clearly support the latter. Whereas the fixed-level masking functions obtained in the previous study showed a dramatic dipper shape and negative masking, the roving-level masking functions obtained here did not. The nonlinear transducer hypothesis predicts equivalent amounts of negative masking with or without a pedestal rove; the intrinsic uncertainty hypothesis, on the other hand, does not. Rather, the intrinsic uncertainty hypothesis predicts no (or attenuated) negative masking under roving-level conditions. We interpret this finding as clear support for the intrinsic uncertainty hypothesis.

This interpretation is predicated on the assumption that one of the consequences of the pedestal rove is to introduce uncertainty about amplitude into the experimental situation, thereby increasing, relative to fixed-level conditions, the number of irrelevant channels monitored by the observer on each trial. As noted in Sec. 1, implicit in this assumption is the notion that these relevant/irrelevant channels are “amplitude channels,” meaning channels tuned to the same frequency but different amplitudes. While, admittedly, it is not as straightforward to conceive of amplitude channels and selective attention thereto as it is to conceive of frequency channels, there is some psychophysical evidence for “attention bands” of this sort (e.g., Luce and Green, 1978), and neurons exhibiting “tuned” responses in the amplitude domain have been observed at various sites in the central auditory nervous system (e.g., primary auditory cortex; Watkins and Barbour, 2011), suggesting that there could be a plausible underlying physiological basis for the effects observed here.

Alternatively, it is possible that uncertainty “about amplitude” effected an increase in the number of monitored channels tuned in some other, nonamplitude domain, and that it was noise in these channels that affected negative masking. Indeed, while research on the effects of uncertainty about amplitude in the context of pure-tone detection and discrimination experiments may be limited, the effects of uncertainty about various other increment parameters [e.g., frequency (Green, 1961) and duration (Dai and Wright, 1995)] are well established. One possibility is frequency. Consider, for example, the “spread of excitation” along the basilar membrane that is elicited by pure-tones and how this spread increases (in terms of its spatial extent) with increasing tone amplitude (e.g., Ruggero, 1992). Consider also how different spatial regions of the basilar membrane are often conceptualized in terms of independent channels tuned to different frequencies. Taken together, these two points suggest that pure-tones with a higher overall amplitude (e.g., *A* ≫ *A_0_*) will stimulate a greater number of frequency channels than those with a lower overall amplitude (e.g., *A* ≤ *A_0_*). What this implies is that, for *A* ≫ *A_0_*, monitoring many frequency channels on each trial may not only have been “not detrimental” in the sense described above with respect to the negligible effect of uncertainty in the suprathreshold region, but rather may have been beneficial in some cases due to the inclusion of channels that may have been more sensitive to changes in the amplitude of the stimulus than the single channel centered on the pedestal frequency (e.g., Zwicker, 1956; Moore and Raab, 1974). For *A* in the region near *A_0_*, however, the spread of excitation presumably was negligible, and more focused attention may have been required to achieve best-possible performance—this being the basic premise of the intrinsic uncertainty hypothesis.

A spread of excitation across frequency channels for *A* ≫ *A_0_*, but not for *A* in the region near *A_0_*, may, in part, explain the lack of negative masking reported here. Recall that, under roving-level conditions, trials in which *A* ≫ *A_0_* were randomly interleaved with trials in which *A* ≤ *A_0_*. Thus, if the observer sought to obtain the best possible performance for suprathreshold pedestals and monitored many frequency channels on each trial, we would expect to find a masking function with a suprathreshold handle evincing the near miss (e.g., Zwicker, 1956; Moore and Raab, 1974) yet no negative masking. In other words, exactly what we found. This interpretation is appealing inasmuch as it provides a unifying account of uncertainty in circathreshold amplitude discrimination in which frequency uncertainty predominates, selective attention in the amplitude domain is not required, and no appeal to amplitude channels is necessary.

Another possible interpretation of the lack of negative masking under roving-level conditions reported here is that the pedestal rove yielded an increase in internal noise in a more general (i.e., not channel-based) sense, as proposed, e.g., in the “context coding” model of amplitude discrimination described by Durlach and Braida (1969; see also Berliner and Durlach, 1973; Berliner *et al.*, 1977). Notably, however, Durlach and Braida's model does not predict differences across pedestal levels in terms of the relative effect of uncertainty, whereas the intrinsic uncertainty hypothesis does. Specifically, the intrinsic uncertainty hypothesis predicts the largest effect of uncertainty for the pedestal level that yields the maximum amount of negative masking.^2^ As discussed in Sec. 3, the results bear this out, and thus are more consistent with the intrinsic uncertainty hypothesis in this respect. It seems unlikely, however, that context effects such as those captured in the Durlach and Braida model had no influence on performance. As such, some combination of these effects and uncertainty should be considered in future work.

## 5. Summary and conclusions

In a previous study (Conroy *et al.*, 2020), we showed that uncertainty induced by random variations in the frequency content of a (nonpedestal) masker can eliminate negative masking in a pure-tone amplitude discrimination experiment. In this study, we showed that uncertainty induced by random variations in the amplitude of the pedestal can do the same. In both cases, uncertainty was conceptualized in terms of a failure of selective attention across channels, tuned either in frequency or in amplitude. Taken together, these two studies suggest that the negative masking of amplitude increments that is often observed in pure-tone amplitude discrimination experiments using circathreshold pedestals is only obtained when “the stars are aligned” and uncertainty about both frequency and amplitude, as well as potentially other stimulus dimensions along which uncertainty may manifest, is at a minimum, consistent with the intrinsic uncertainty hypothesis.

## Footnotes

^1^ While our focus in this article is audition, it should be noted that negative masking is a general phenomenon that has been reported in multiple sensory modalities (see Solomon, 2009, for a review).
